# Assessment of cross-protection induced by a bluetongue virus (BTV) serotype 8 vaccine towards other BTV serotypes in experimental conditions

**DOI:** 10.1186/s13567-018-0556-4

**Published:** 2018-07-16

**Authors:** Ludovic Martinelle, Fabiana Dal Pozzo, Christine Thys, Ilse De Leeuw, Willem Van Campe, Kris De Clercq, Etienne Thiry, Claude Saegerman

**Affiliations:** 1 Faculty of Veterinary Medicine, Research Unit in Epidemiology and Risk Analysis Applied to Veterinary Sciences (UREAR-ULg), Fundamental and Applied Research for Animal and Health (FARAH) Center, University of Liege, Liege, Belgium; 2 Sciensano, Exotic Viruses and TSE, Ukkel, Belgium; 3 Sciensano, Experimental Centre, Machelen, Belgium; 4 Faculty of Veterinary Medicine, Fundamental and Applied Research for Animal and Health (FARAH) Center, Veterinary Virology and Animal Viral Diseases, University of Liege, Liege, Belgium

## Abstract

Bluetongue disease is caused by bluetongue virus (BTV) and BTV serotype 8 (BTV8) caused great economic damage in Europe during the last decade. From 1998 to 2007, in addition to BTV8, Europe had to face the emergence of BTV1, 2, 4, 9, and 16, spreading in countries where the virus has never been detected before. These unprecedented outbreaks trigger the need to evaluate and compare the clinical, virological and serological features of the European BTV serotypes in the local epidemiological context. In this study groups of calves were infected with one of the following European BTV serotypes, namely BTV1, 2, 4, 9 and 16. For each tested serotype, two groups of three male Holstein calves were used: one group vaccinated against BTV8, the other non-vaccinated. Clinical signs were quantified, viral RNA was detected in blood and organs and serological relationship was assessed. Calves were euthanized 35 days post-infection and necropsied. Most of the infected animals showed mild clinical signs. A partial serological cross reactivity has been reported between BTV8 and BTV4, and between BTV1 and BTV8. BTV2 and BTV4 viral RNA only reached low levels in blood, when compared to other serotypes, whereas in vitro growth assays could not highlight significant differences. Altogether the results of this study support the hypothesis of higher adaptation of some BTV strains to specific hosts, in this case calves. Furthermore, cross-protection resulting from a prior vaccination with BTV8 was highlighted based on cross-neutralization. However, the development of neutralizing antibodies is probably not totally explaining the mild protection induced by the heterologous vaccination.

## Introduction

Bluetongue virus (BTV) represents the type species of the *Orbivirus* genus, family *Reoviridae* and causes bluetongue disease (BT) in susceptible species [1, 2]. BTV is usually transmitted to domestic and wild ruminants by the bite of haematophagous female midges of the *Culicoides* genus yet direct transmission was demonstrated at least for serotype 26 [3]. From 1998 to 2006, Europe had to face an unprecedented emergence of BTV serotypes 1, 2, 4, 9 and 16 (BTV1, 2, 4, 9, 16) throughout the Mediterranean Basin, including several countries where the virus was never detected before. August 2006 is a tipping point in BTV epidemiology, with a first detection of BTV8 in Europe Mainland [4] and a subsequent wide spread throughout Europe during the following 2 years. BTV8 emergence was easily spread through Culicoides species that were not known as the historic BTV transmission species, i.e. *Culicoides obsoletus* complex species [5]. This epidemic—affecting abundantly cattle whereas previous outbreaks largely occurred in small ruminants—is considered to have caused greater economic damage than any previous single serotype outbreak [6]. Most of the countries involved in the beginning of the BTV8 epidemic and that paid the heaviest toll were declared bluetongue-free in 2012 (Belgium, the Netherlands, Germany, France [7, 8]).

Bluetongue virus virulence and transmission potential is not serotype driven thus outcome of the infection cannot be predicted based on the serotype alone [9]. Within a serotype, the geographical origin can be used to define topotypes with different pathogenicity. As an example, some Australian strains were reported to be less virulent than their Western counterparts [10]. The presence of competent palearctic vectors and several serotypes recently described in Europe mainland, with non-immunized livestock, trigger the need to evaluate and compare the clinical, viral and immunological features of the European BTV serotypes in cattle. In addition, since the European BTV8 showed an unusual virulence in cattle, the emergence of another serotype could take place in an area with local cattle possibly already immunized against BTV8.

Serological relationships between the different BTV serotypes were mostly established more than 25 years ago based on plaque reduction tests and cross-protection experiments in sheep [11]. It is assumed that there is partial or no cross-protection between the different BTV serotypes, therefore the need of serotype specific vaccination strategies. At the moment, a total of 27 serotypes have been recorded [12], possibly 29 [13]. As a consequence, developing and implementing multi-serotype prophylactic approaches to tackle BTV is one of the major challenges in the control of the disease. Cross-reactivity between BTV1 and BTV23 [14], BTV1 and BTV8 [15] or more recently between BTV16 and multivalent serum of sheep vaccinated against BTV9, 2 and 4 [16] was reported. These serotypes are however traditionally considered as poorly related.

The current study was implemented to pursue two main objectives. First, to assess and compare the virulence of some of the BTV serotypes threatening Europe mainland—namely BTV1, BTV2, BTV4, BTV9 and BTV16—in controlled conditions in calves. Second, to evaluate the extent of cross-protection granted by BTV8 vaccination in calves infected with these serotypes. In addition, in vitro humoral cross-reactivity was determined.

To these ends, each of the tested serotypes (BTV1, BTV2, BTV4, BTV9, and BTV16) was used to infect two groups of calves. One group was vaccinated against BTV8 using a commercial inactivated vaccine, and the other group was not. The clinical, pathological and virological consequences of the infection with these different serotypes, whether the animals were vaccinated or not, were compared, and serological relationships were assessed.

## Materials and methods

### Animals

Animals were treated in accordance with the International Guiding Principles for Biomedical Research Involving Animals, as issued by the Council for the International Organizations of Medical Sciences and EU Directive 2010/63/EU for animal experiments.

A total of 35 Holstein male calves, about 5.5–6 months old, were used. All the selected animals were tested seronegative (ELISA and seroneutralization) and non viraemic (RTqPCR) for BTV and Bovine herpesvirus 1 (BoHV1). In addition these calves were also born from BTV naïve dams (seronegative and RTqPCR negative). They were introduced in an insect secured BSL3 facility 1 week before the beginning of the experiment to allow their acclimatization.

### Virus

BTV1, BTV2, BTV4, BTV9 and BTV16 were all derived from the reference strains of the Onderstepoort Veterinary Institute. These strains underwent further passages at The Pirbright Institute (TPI); passage history is available at the RNAs and Proteins of dsRNA Viruses [17]. The BTVs were subsequently passaged at Sciensano, Ukkel, Belgium (formerly CODA–CERVA) between two and four times on BHK-21 cells. BTV8 originated from a field sample (BEL2006/01) afterwards passaged 6 times in BHK-21. Each serotype has been tested by RTqPCR specific of the serotypes used in the study to rule out potential contamination of the inocula.

### Experimental design

Five animals were kept as environmental control, and were inoculated with Dulbecco’s Modified Eagle Medium (DMEM, Life Technologies, Gent, Belgium). Groups were identified by their vaccination status against BTV8 (V_ or NV_, respectively vaccinated and non-vaccinated) followed by the subsequently inoculated BTV serotype (BTV1, BTV2, BTV4, BTV9 or BTV16). For each tested serotype, two groups of three calves each were used. The animals from the first group were vaccinated against BTV8 (BTVPUR AlSap 8, Merial, Lyon, France) following manufacturer instructions, with the second vaccine injection 33 days before challenge. In the other group the animals were not vaccinated.

To be infected, the animals received between 2.5 and 4 mL of inoculum, properly diluted to a normalized titre of 10^6^ TCID_50_/animal. Inoculations were realised through the subcutaneous route, on the left side of the neck. Daily examination of the calves included temperature and clinical signs monitoring for 35 days post-infection (dpi). The severity of the infection was quantified by calculating clinical scores per system and per animal, leading to overall clinical scores by groups and animal, following a standardised clinical form adapted from Saegerman et al. [18]. Briefly, clinical signs were summed up according to their nature (general signs versus localised clinical signs on muzzle, mouth, limbs and eyes) and intensity (crust, ulcerations or necrosis, oedema or inflammation). The calves were euthanatized at 35 dpi by captive bolt stunning followed by bleeding. Extensive necropsy has been performed, and spleen, thymus, prescapular and mesenteric lymph nodes, testicle and lung were sampled from infected and control calves, to detect BTV RNA by RTqPCR.

### BTV RNA detection

Viral RNA extraction from the blood was achieved using the QIAamp Viral RNA Mini Kit (Qiagen, Antwerp, Belgium). In the organs, about 100 mg of tissues per organ were processed; viral RNA extraction was performed using Trizol reagent according to the manufacturer’s instructions (Gibco Invitrogen, UK). Viral RNA denaturation and reverse transcription were adapted from previously published protocols [19] with slight modifications, as denaturation was realised in presence of random hexamers. Serotype specific RTqPCR assays were carried out for BTV1, BTV2, BTV4, BTV8, BTV9 and BTV16 using LSI VetMAX European BTV Typing Real-Time PCR Kits (ThermoFisher Scientific, Gent, Belgium), following manufacturer’s instructions.

RTqPCR reactions were run on a CFX96 Touch™ Real-Time PCR Detection System (Bio-Rad Laboratories N.V., Temse, Belgium) using the following cycling conditions: heat inactivation at 95 °C for 10 min, 50 cycles consisting of denaturation at 95 °C for 15 s and annealing/elongation at 58 °C for 30 s.

To allow absolute quantification of the viral RNA content in blood and organ samples, standard curves of serotype specific plasmids (pGEM^®^-T Easy Vector, Promega, The Netherlands), carrying the target part of the segment 2, were constructed. Quantification was expressed in cDNA copy number/mL of blood.

### Anti-BTV antibodies detection

For each tested serotype neutralizing antibodies (Abs) were titrated by seroneutralization (SNT). Two-fold serial dilutions of the sera (1:10–1:1280) were tested in the presence of 100 TCID_50_ of BTV, as previously described [20]. The neutralizing antibody titre was defined as the reciprocal of the serum dilution causing a 50% reduction in cytopathic effect. Serum of all the animals has been tested at several time points with the homologous virus.

Anti-VP7 antibodies circulation was also evaluated using a commercial competitive ELISA kit (ID Screen^®^ Bluetongue Competition ELISA kit, ID Vet, France). Results were expressed as % negativity (PN) compared to the negative kit control and transferred to a positive, doubtful or negative result according to the cut-off settings provided by the manufacturer (PN ≤ 35 is positive; 35 < PN ≤ 45 is doubtful; PN > 45 is negative). As these cut-off values were rather designed for screening purposes [21], the cut-off suggested by Vandenbussche et al. [19] (negative when PN > 66) has been also considered as a tool for individual diagnostic, with respect of the limited number of animals.

### Haematology

Starting during the acclimatization period and until the end of the experiment, a complete haemogram (Vet ABC, SCIL animal care company, France), including total leukocytes, monocytes, lymphocytes, neutrophils, eosinophils and basophils was performed on EDTA blood samples on a regular basis.

### Cross-neutralization assay

In each non-vaccinated group, the individual serum sample with the highest homologous neutralizing titre has been selected and subsequently tested by SNT against all the other inoculated BTV serotypes. In order to avoid potential bias due to low humoral response against any of the serotype, heterologous neutralization results for each tested serotypes were expressed as a percentage of the titre reached when the immunised serum was tested with the homologous serotype. BTV8 immune serum was obtained from an experimentally infected heifer in a previous study [22].

### In vitro kinetic growth of BTV serotypes

In vitro growth properties of the 6 BTV serotypes (i.e. BTV1, BTV2, BTV4, BTV8, BTV9 and BTV16) used in this study were compared using their replication kinetics in VERO cell culture, following a protocol adapted from Dal Pozzo et al. [23]. Briefly, all the inocula were used in a one-step growth assay, with confluent VERO cells, at a multiplicity of infection (m.o.i.) of 0.05. After 0, 8, 24, 48, 72, 96 and 120 h incubation, the supernatant was removed and stored −80 °C. For each time point, the virus titre was determined at least in triplicate by plaque assay [24] and expressed as Log TCID_50_/mL.

### Infectivity

For each serotype, the original inoculum plus two serial 1:10 dilutions were tested by RTqPCR. Knowing the infectious titre of each inoculum, for each serotype a mean ratio of segment 2 (S2) cDNA/TCID_50_ was then calculated. The infectious titre of the blood samples was then extrapolated using RTqPCR results multiplied by the mean segment 2 cDNA/TCID_50_ ratio and expressed as TCID_50_/mL, for each tested time points. TCID_50_/mL titres were then converted in PFU/mL to assess the level of infectivity of each serotype based on the estimate of the minimal PFU/mL required to infect vector *Culicoides* according to Dungu et al. [25].

### Statistical analysis

For viraemia levels and infectivity assessment between vaccinated and non-vaccinated groups, the comparison of quantitative parameters was performed using pair-wise t tests or Welch test, as appropriate. One-way ANOVA with post hoc Tukey test were used to analyse haematological values within the same group with respect to baseline values at 0 dpi. Differences between control and infected groups at the same time-point were analyzed using a two-way mixed model ANOVA with Bonferroni post-test. Two-way mixed model ANOVA were calculated using the statistical analysis program GraphPad Prism version 5.01 for Windows (GraphPad Software, San Diego California USA). Other statistical analyses were realized using the R software/environment (R-3.2.1, R Foundation for Statistical Computing). For all tests, *P* values < 0.05 were considered significant.

## Results

### Clinical examination

**Figure 1:**
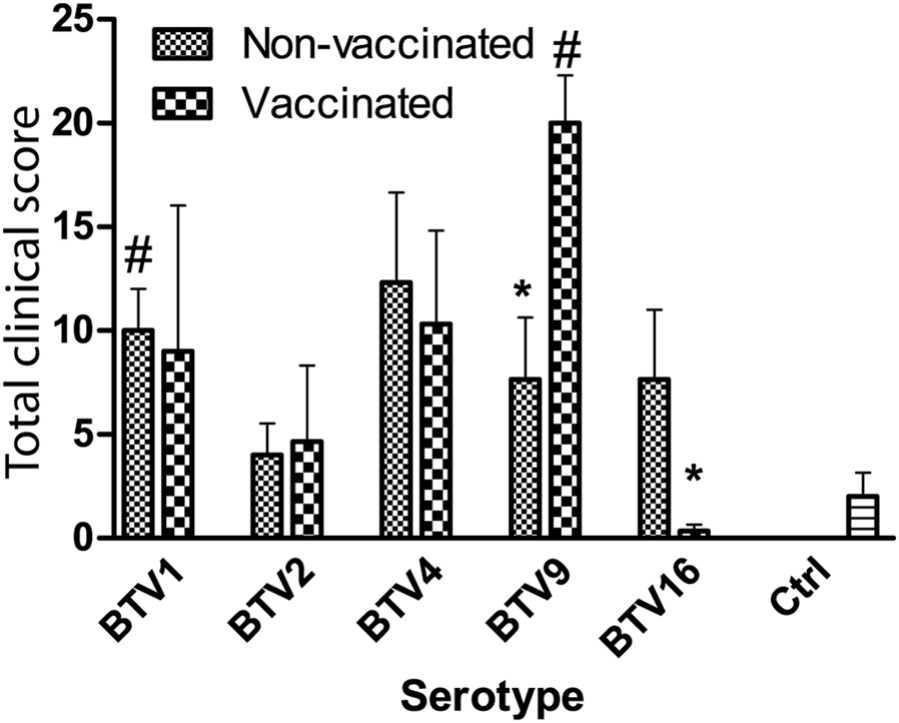
**Total clinical scores by group and vaccination status.** Error bars represent standard error of the mean. *P < 0.05 between vaccinated and non-vaccinated groups infected with the same serotype. ^#^*P* < 0.05 compared to the control group.

Two calves from the control group showed 1–2 days-lasting hyperthermia between 6 and 12 dpi, without other systemic problem or clinical signs evocative of bluetongue disease. In infected animals, clinical signs were mild, and would probably go unnoticed in the field, as appetite was conserved and general condition unchanged. Nevertheless, typical bluetongue clinical signs were observed, including facial oedema, swelling and reddening of the odontoid papillae, crusts and erosions at the muco-cutaneous junction, nasal discharge and purulent conjunctivitis. V_BTV9 group and NV_BTV1 group clinical scores were significantly higher than control group (*P* < 0.05). V_BTV9 and NV_BTV16 groups had a significantly higher clinical score than their counterparts infected with the same serotype but different vaccination status. There were no significant differences between clinical scores of other infected groups when compared to each other, to the control group or when comparing groups infected with the same serotype but with different vaccination status (Figure 1). A great individual variability was observed in the clinical outcomes within each group, as in 6 out of 10 infected groups, one single animal totalized 50% or more of the total clinical score of the group.

### BTV RNA detection

**Figure 2:**
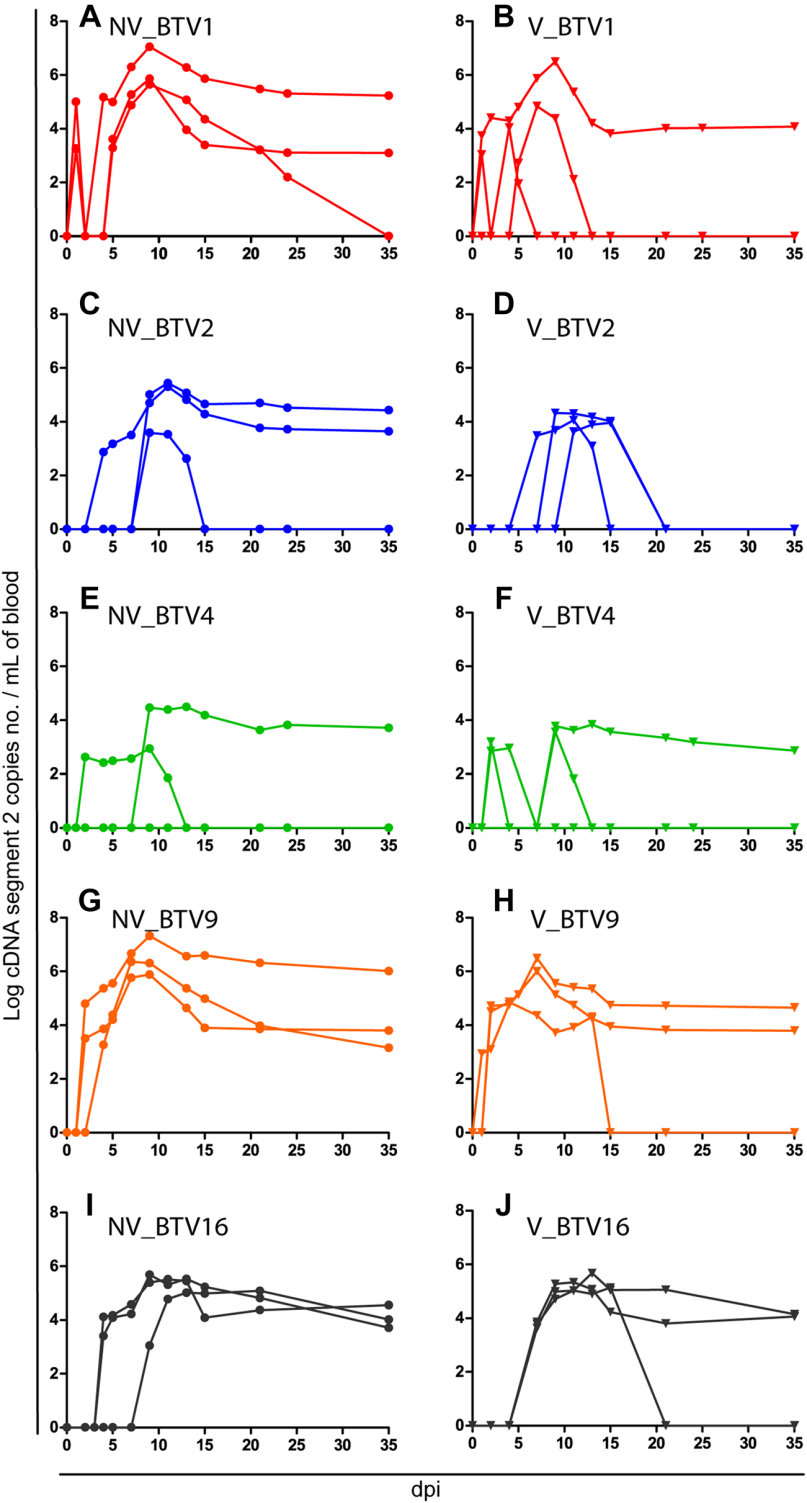
**Individual daily viral genome load in calves’ blood.** Results are expressed as the Log copies of BTV segment 2 cDNA per mL of blood. **A** and **B** BTV1; **C** and **D** BTV2; **E** and **F** BTV4; **G** and **H** BTV9; **I** and **J** BTV16. **C**, **E**, **G** and **I** are non-vaccinated groups whereas **B**, **D**, **F**, **H** and **J** are vaccinated groups; dpi: day post-infection.

No viral RNA was detected in any of the control animals at any time point. Inoculated animals showed different viraemia patterns depending on the inoculated serotype and their vaccination status. The earliest BTV RNA detection in the blood occurred at 1 dpi in NV_ and V_BTV1 groups. The latest onset of viraemia occurred in one calf of V_BTV2 group at 11 dpi. In calves infected with serotypes 2 and 4 BTV RNA could only be detected inconsistently and RNAemia reached moderate levels when compared to calves infected with BTV1, 9 and 16 (Figure 2). After the challenge of vaccinated animals, the Log copy number of viral RNA was significantly lower in BTV2 and BTV4 groups when compared to BTV9 and BTV16 groups (Two way ANOVA with repeated measures, *P* < 0.004). In addition, BTV1 RNA detection was also significantly lower than BTV9 (*P* < 0.013). After the challenge of non-vaccinated animals viral RNA detection was significantly lower in BTV2 and BTV4 groups when compared with BTV9 and BTV1 groups (*P* < 0.003), and BTV4 RNA detection was significantly lower than BTV16 (*P* < 0.005). Regarding homologous serotypes, only BTV1 showed a lower RNA detection in V group versus NV group (*P* < 0.016). Vaccinated animals had an RNAemia ranging from 79.4 to 95.5% of the max RNAemia level of the non-vaccinated animals at the viraemic peak. At the end of the experiment viral RNA was still detectable in 40% of the vaccinated animals versus 73% in the non-vaccinated calves. However this difference was not significant (χ^2^, *P* = 0.065).

### Anti-BTV antibodies detection

**Figure 3:**
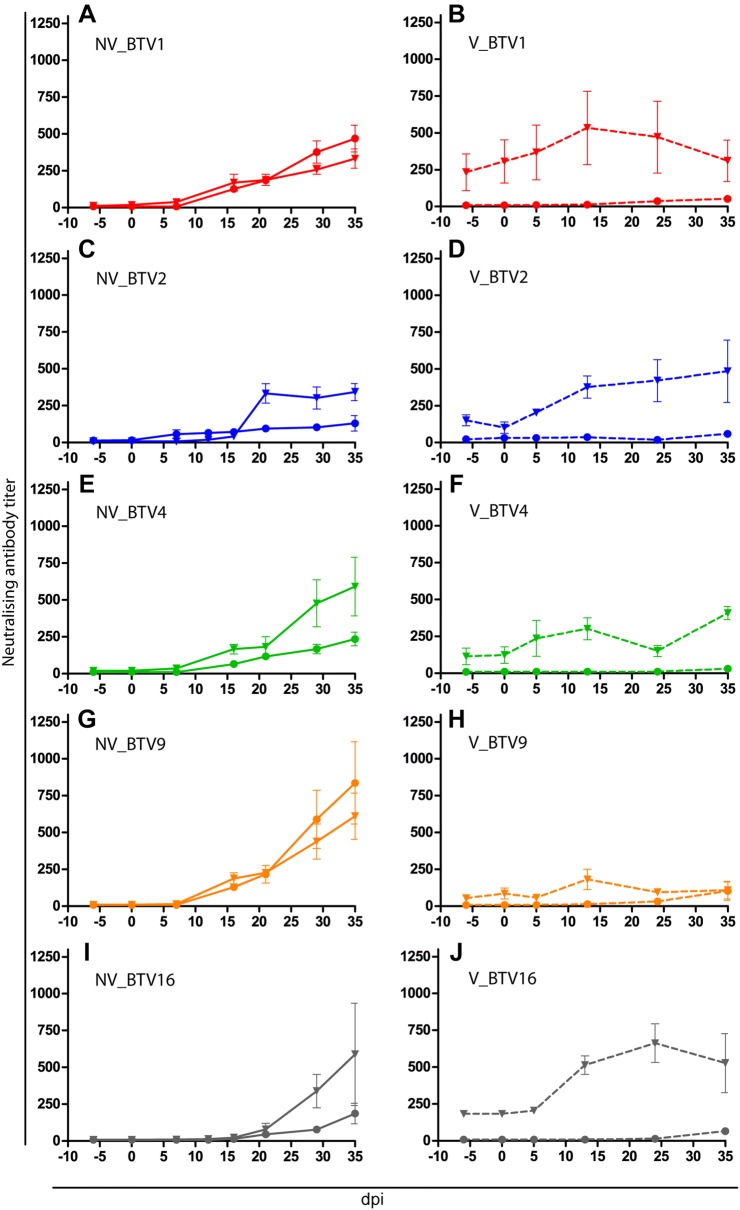
**Evolution of neutralising antibodies titres against BTV8 and homologous inoculated serotypes. A**, **C**, **E**, **G** and **I** (full lines): mean neutralising antibodies titres per group against respectively BTV1, 2, 4, 9 and 16. In each panel vaccinated (triangle) and non-vaccinated (filled circle) groups are represented. **B**, **D**, **F**, **H**, **J** (dashed lines): mean neutralising antibodies titres against BTV8 in vaccinated (triangle) and non-vaccinated (filled circle) animals in respectively BTV1, 2, 4, 9 and 16 inoculated groups. Error bars represent standard error of the mean. dpi: day post-infection.

All the inoculated animals produced homologous neutralizing antibodies that started generally to be detected two to 3 weeks post-infection (Figure 3). In previously BTV8 vaccinated calves, the rise of neutralizing antibodies against the virus serotype used in the challenge was earlier detected and reached higher levels compared to non-vaccinated calves however only significant for BTV2 infected animals (*P* < 0.035). In vaccinated animals, anti-BTV8 neutralizing antibodies were contemporaneously circulating (Figures 3B–J) and anti-BTV8 titres were significantly higher than in NV animals for all serotypes. Whereas no significant increase in anti-BTV8 neutralizing antibodies could be detected in non-vaccinated animals, vaccinated ones from groups inoculated with BTV2 and 16 underwent a slight boost in anti-BTV8 neutralizing antibodies titres, despite the heterologous nature of inoculated serotype (*P* < 0.006).

Neutralizing antibodies titres had no correlation with viral RNA detection level (r = 0.12, *P* = 0.11) and maximal SNT titres had no correlation with maximal BTV RNA copy number (r = 0.09, *P* = 0.63).

**Figure 4:**
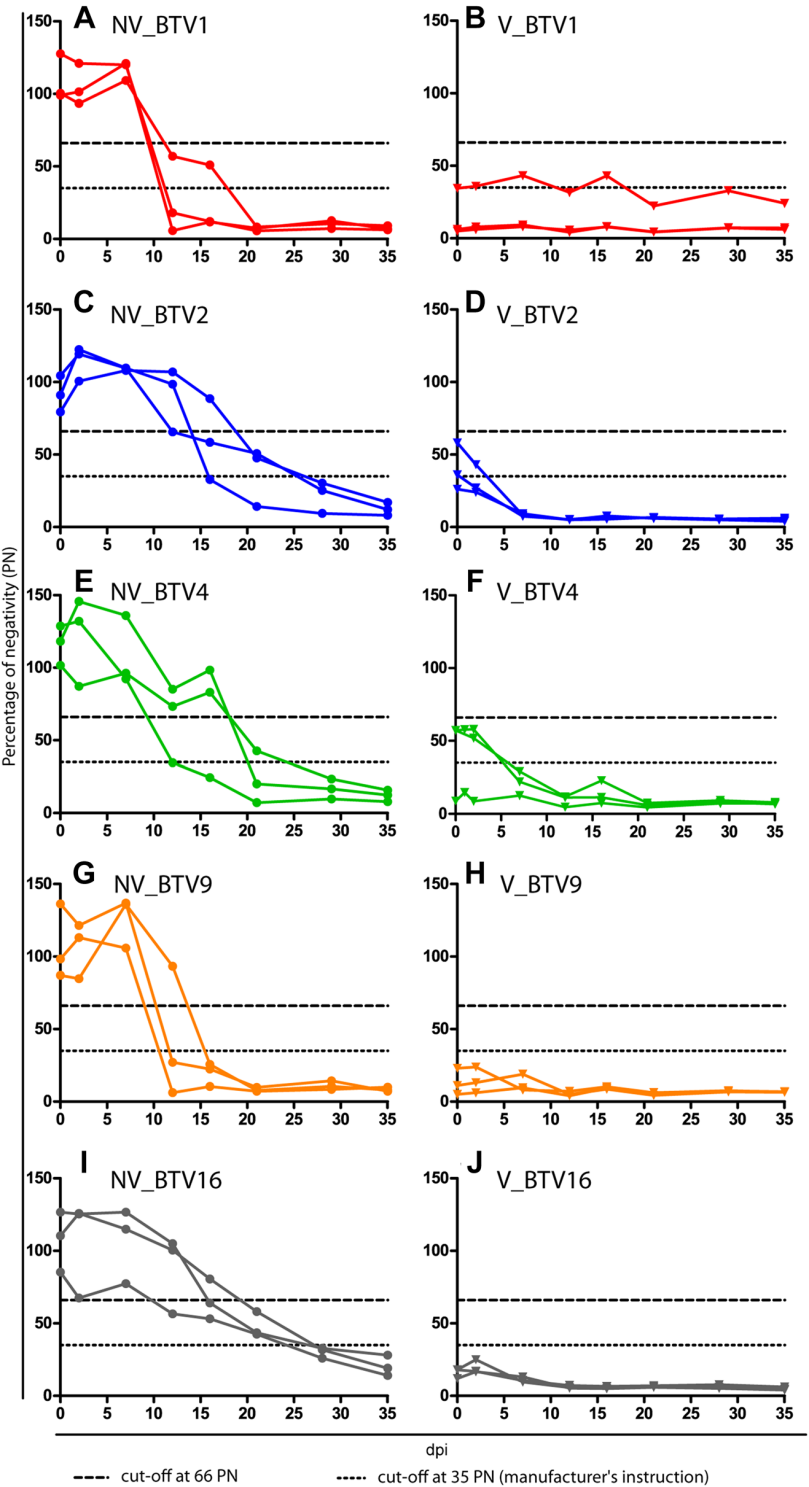
**Evolution of serogroup specific antibodies.** The results are presented for each animal as the percentage of negativity (PN) obtained in the competitive ELISA. Data for non-vaccinated (filled circle) and vaccinated (triangle) animals are shown for BTV1 (**A**, **B**), BTV2 (**C**, **D**), BTV4 (**E**, **F**), BTV9 (**G**, **H**) and BTV16 (**I**, **J**). Dotted line represents the cut-off value recommended by the manufacturer and dashed line the one suggested by Vandenbussche et al. [18]. dpi: day post-infection.

The cELISA kit managed to detect Anti-VP7 antibodies for each tested serotype. The time of seroconversion was in line with previously published data [20, 26, 27], between 10 and 21 dpi for all serotypes (Figure 4). By the time of inoculation, 2 V_BTV2 and 2 V_BTV4 calves had a PN above the positivity threshold (thus considered negative) determined by the manufacturer (PN ≤ 35), however considered positive if taking into account of the threshold defined by Vandenbussche et al. [19] (PN ≤ 66). All these 4 animals had PN that reached values under 35 within 7 dpi.

### Haematology

**Figure 5:**
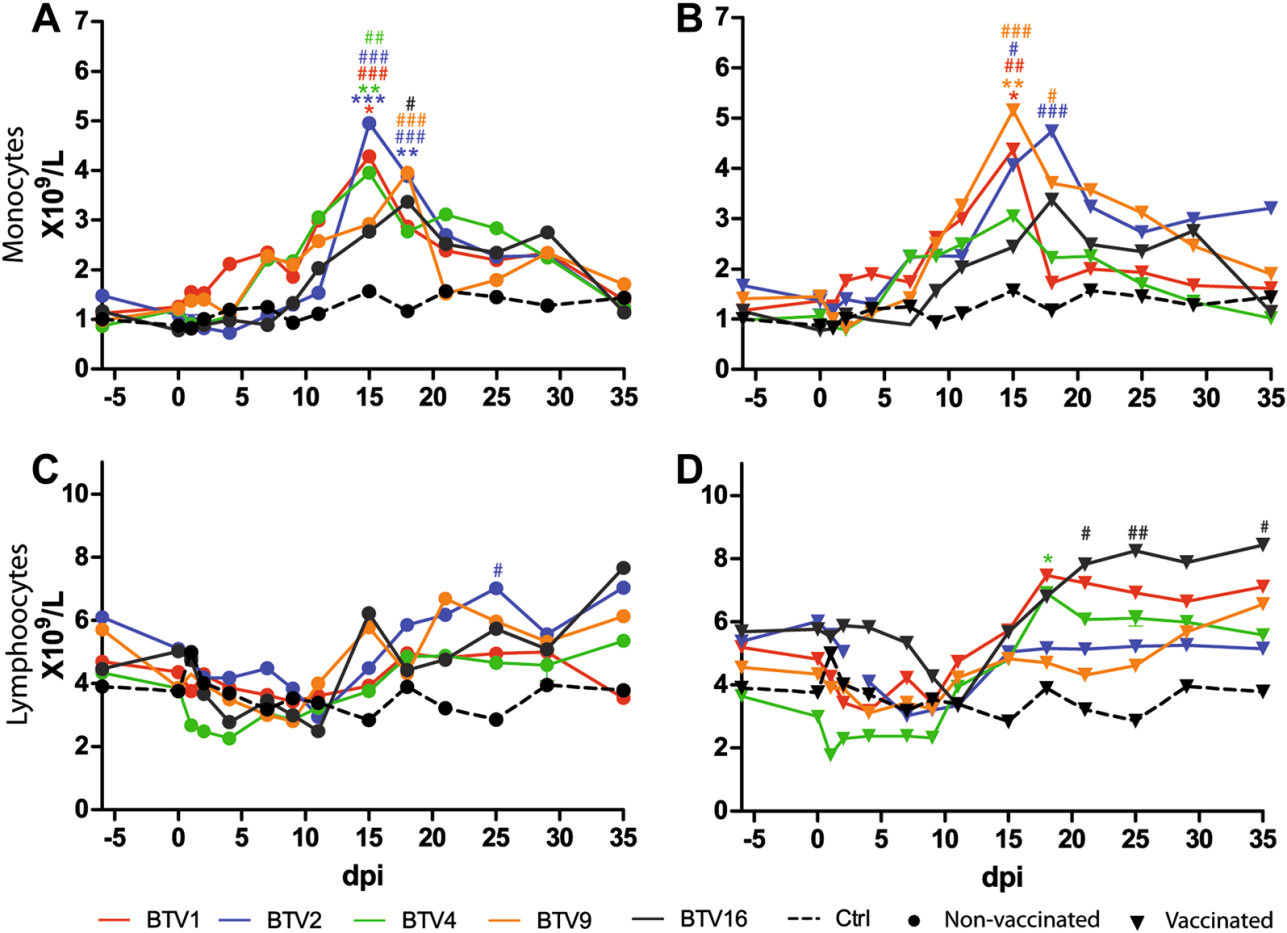
**Lymphocytes and monocytes cell counts.** Mean monocytes (**A** and **B**) and lymphocytes (**C** and **D**) cell counts in non-vaccinated (**A** and **C**) and vaccinated animals (**B** and **D**), expressed as 10^9^ cells/L. dpi: day post-infection. For a better readability error bars were removed. *P < 0.05; **P < 0.01 and ***P < 0.001 between vaccinated and non-vaccinated groups infected with the same serotype. ^#^*P* < 0.05; ^##^*P* < 0.01; ^###^*P* < 0.001 compared to the control group.

Monocyte counts increased shortly after infection in all groups but control animals independently of their vaccination status. The increase started between 4 and 11 dpi, peaked at 15 or 18 dpi and then came back to baseline levels by 35 dpi (Figure 5). All NV groups showed peaks significantly higher than baseline value (0 dpi) or control value at the same time point (Figure 5A, *P* < 0.05, two way ANOVA with repeated measures) whereas in V groups only peaks of V_BTV1, BTV2, and BTV9 were significantly higher (Figure 5B, *P* < 0.05, two way ANOVA with repeated measures).

Lymphocyte count followed a different trend, decreasing in the first dpi, from about 4–11 dpi, then recovering and even significantly exceeding baseline values (Figure 5D, V_BTV4: 18 dpi, *P* < 0.05, two way ANOVA with repeated measures) or control values at the same time point (Figures 5C and D, NV_BTV2: 25 dpi and V_BTV16: 21, 25 and 35 dpi, *P* < 0.05, two way ANOVA with repeated measures).

### Necropsy and BTV RNA detection in organs

At the necropsy, lesions were sporadically reported, including haemorrhage and petechial haemorrhage on several lymph nodes, endocardic suffusion, abscesses and petechial haemorrhage in the lung, and a slight haemorrhage in the wall of the pulmonary artery of one calf in the NV_BTV9 group.

No viral RNA could be detected in any organs of the animals of the BTV2 and BTV4 groups, whether they were vaccinated or not (Table 1). The proportion of positive organs was significantly higher in BTV9 infected groups (pairwise Fisher test, *P* < 0.002). However there was no significant difference between BTV9 V and NV groups regarding organ detection (*P* = 0.075). Considering all serotypes all together, viral RNA was most commonly detected in prescapular lymph node (Table 1). In vaccinated animals infected with BTV9, detection of BTV RNA in organs was associated with an Odds Ratio of 0.16 (*P* < 0.03, [0.029–0.691]).

**Table 1:** **BTV RNA detection in organs at necropsy**

	Calf ID	Spleen	Mesent. LN	Prescap. LN	Thymus	Testicle	Lung
NV_BTV1	169	NEG	NEG	7.41	NEG	NEG	NEG
	1004	NEG	NEG	6.27	NEG	3.33	NEG
	6712	NEG	NEG	4.92	NEG	NEG	NEG
V_BTV1	2038	NEG	NEG	0.40	NEG	NEG	NEG
	2044	1.42	NEG	18.96	NEG	NEG	NEG
	5093	NEG	NEG	NEG	0.15	NEG	NEG
NV_BTV9	1058	NEG	4.73	57.43	28.61	2.23	0.57
	2071	12.22	4.36	18.78	NEG	1.89	1.30
	3045	2.00	1.14	5.72	0.52	4.59	NEG
V_BTV9	2740	NEG	7.40	NEG	24.48	1.71	NEG
	3934	0.96	0.88	2.09	NEG	NEG	NEG
	4935	11.84	NEG	9.46	NEG	NEG	NEG
NV_BTV16	5583	NEG	NEG	2.25	NEG	NEG	NEG
	8606	NEG	NEG	NEG	NEG	NEG	NEG
	9535	NEG	0.23	NEG	17.34	NEG	NEG
V_BTV16	2461	NEG	NEG	NEG	NEG	NEG	NEG
	3150	NEG	NEG	NEG	97.22	NEG	NEG
	5077	NEG	NEG	2.70	NEG	NEG	NEG

Results are expressed a BTV RNA copy number per 100 mg of tissue.

No positive detection could be found in any tested sample in BTV2 and BTV4 vaccinated or non-vaccinated groups.

NV_: non vaccinated group; V_: vaccinated group; NEG: negative result; Mesent. LN: mesenteric lymph node; Prescap. LN: prescapular lymph node.

### Cross neutralization assay

Different degrees of in vitro cross neutralization could be found using all immunized sera (Table 2). However, immunized sera of BTV2 and BTV9 showed the least degree of in vitro cross neutralization against the other tested viral serotypes. On the contrary, BTV8 immune serum had a higher neutralization effect in vitro on the growth of BTV4, reaching a titre equal to 25% against BTV4 when compared to the titre reached against BTV8 itself. BTV1 immune serum reached a similar level of partial seroneutralization against BTV8. A lesser cross neutralization has been reported with the BTV16 serum towards BTV1 virus (Table 2).

**Table 2:** **Percentages of relative homologous and heterologous seroneutralization titers**

Immunised serum	BTV serotype					
	BTV1	BTV2	BTV4	BTV8	BTV9	BTV16
Control	0.52	1.11	0.55	0.44	0	3.54
BTV1	100	1.11	8.85	25	0	3.54
BTV2	1.3	100	3.15	0.44	0	3.54
BTV4	0.65	0.88	100	0.44	0	12.5
BTV8	10.36	12.5	25	100	6.25	3.54
BTV9	2.61	3.1	0.77	0.44	100	3.54
BTV16	18.34	12.39	0.83	0.44	0	100

### In vitro kinetic growth of BTV serotypes

**Figure 6:**
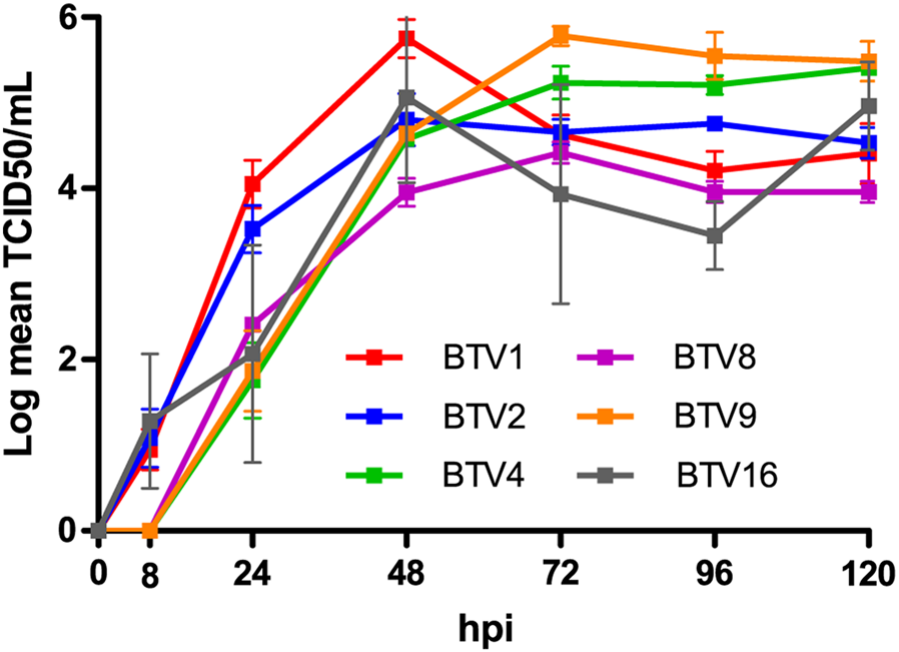
**In vitro kinetic growth of BTV1, 2, 4, 9, and 16.** Growth curves of BTV1, 2, 4, 8, 9 and 16 in VERO cells. Cells were infected at a MOI of 0.05 and supernatant collected at 8, 24, 48, 72, 96 and 120 h post-infection (hpi). Then supernatants were titrated on VERO cells by end-point dilution assay and expressed as the Log (TCID_50_/mL). Each growth curve has been established independently at least in triplicate for each serotype.

The mean virus titres measured for each serotype at the different time points were compared to all the other serotypes using a two-factor ANOVA with repeated measures on one factor, showing no significant difference between the different kinetic growth curves (*P* = 0.41, Figure 6).

BTV1, 2, 6 and 16 had earlier replication as cytopathic effect (CPE) has been reported since 8 h post-infection (hpi), whereas BTV4, 8 and 9 showed CPE starting at 24 hpi.

### Infectivity

**Figure 7:**
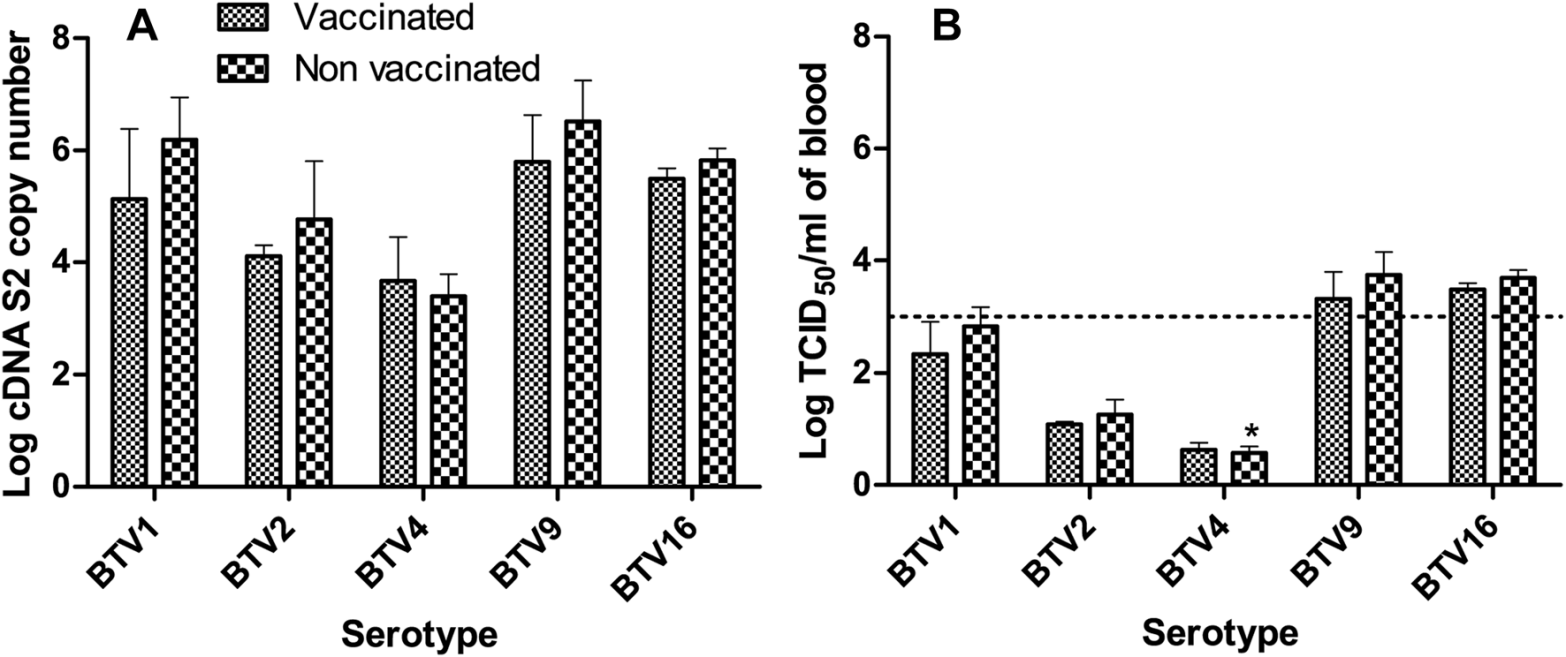
**Mean S2 cDNA copy number and infectivity at viraemic peak.** **A** S2 cDNA mean copy number at viraemic peak by tested serotype and vaccinated status. **B** Infectivity at viraemic peak, expressed as mean Log TCID_50_/mL of blood titre by tested serotype and vaccinated status. Dotted line represents the classical threshold of 10^3155^ TCID_50_/mL required to allow BTV transmission to vector *Culicoides*. *For NV_BTV4, one animal had no detectable viraemia at all was excluded from calculation. Error bars represent standard deviation.

At viraemic peak S2 copies number ranged from 10^6.52 (± 0.73)^ for NV_BTV9 to 10^3.39^ ^(± 1)^ for NV_BTV4 (Figure 7A), which correspond to a titre of 10^3.74 (± 0.42)^ TCID_50_/mL for NV_BTV9 group and 10^0.58 (± 0.11)^ TCID_50_/mL for NV_BTV4 (Figure 7B). Only BTV9 and BTV16 groups had consistently max viraemia higher than 10^3155^ TCID_50_/mL (threshold value under which oral infection of *Culicoides* is supposed to be impossible according to Dungu et al. [25]) in all animals. Amongst BTV1 infected calves only NV_BTV1 calf 0169 had a max viraemia above the threshold. No animals in the BTV2 or BTV4 groups had a viraemia above 10^3155^ TCID_50_/mL whichever was the considered time point or vaccination status.

## Discussion

In this study BTV inoculation resulted from asymptomatic affection to mild clinical signs. Although controversial [20, 28, 29] the passage history of the virus used in the current study could influence the clinical expression of the disease. Individual susceptibility could be another explanation [1].

Unlike in many other viral diseases [30–32], it was not possible to demonstrate a statistically significant relation between level and duration of viraemia, and clinical presentation. However this is not surprising in BTV infection, as RNAemia lasts longer in cattle than in sheep despite the more severe outcome in the latter species [33]. Furthermore, BTV clinical picture in cattle is usually subtle and does not allow to make any correlation with the viral RNA detection in the blood.

On the other hand, BTV replication level has been correlated to the adaptation of the virus to the host [34]. In this study and based on the levels of viral RNA detection serotypes were ranked as follows from the most to the less adapted in calves: BTV9, 1, 16, 2 and 4 in non-vaccinated groups, and BTV9, 16, 1, 2 and 4 in vaccinated ones. The limited circulation of BTV2 and BTV4 RNA in blood might explain the absence of positive detection in organs at necropsy. The hereinabove suggested host adaptation ranking based on BTV serotypes might involve non-serotype specific virulence factors, in line with findings from Caporale et al. [35].

For standardization purposes the same doses were inoculated, regardless of the serotype. It has to be stressed that different BTV strains or serotypes, as a consequence of their large variability, their origin, could indeed be non-equivalently adapted to their hosts. Dal Pozzo et al. described a dominance of BTV8 in cattle when compared to BTV1 and BTV15 [23]; North American serotype 10 replicates more efficiently in sheep than serotype 17 [36], and comparing BTV1 and BTV15, BTV1 seems to be better adapted to sheep whereas BTV15 is better adapted to cattle [34]. In addition, the modified live vaccine (MLV) required doses to grant protection in sheep have been determined to be different depending on the considered serotype [37]. Indeed the low replication level of BTV4 and BTV2 observed in this study might rely on an inoculation dose insufficient for these serotypes and support the hypothesis of a poor adaptation to cattle.

Amongst the serotypes used in this study, Erasmus described serolological relationship, based on cross-protection tests in sheep or heterotypic antibody responses, between serotypes 2 and 1, 1 and 9, and 9 and 4; serotypes 8 and 16 being quite serologically isolated [11]. In the current study cross neutralization tests revealed moderated antigenic relationships mainly between serotypes 4 and 8, 1 and 8 and to a lesser extent 2 and 9. Partial cross neutralization has been previously reported between serotypes 1 and 8 [15]. The discrepancies among the above mentioned studies have to be interpreted carefully, as the origin of the used isolates varies greatly, from African strains to European field isolates. In addition, the intra-serotype VP2 nucleotide sequence variation can be up to more than 30% [38]. The quasispecies nature of BTV, its evolution through genetic drift and founder effect could explain the divergences between VP2 amino acids sequences and in vitro cross neutralization assays.

In this study the serological cross-reactivity with BTV8 was limited to BTV4 and BTV1. Consequently, the differences observed in the course of the current experiment between vaccinated and non-vaccinated groups are most likely unrelated to humoral immunity. Numerous studies have described the importance of cell-mediated immunity in the course of BTV infection [14, 39]. Cell-mediated immunity is suggested to rely to an important extent on non-structural proteins [40]. Despite non-structural proteins not being part of the viral particles used to produce inactivated vaccines, vaccinated animals were reported to develop antibodies against NS proteins [41]. Therefore, in this study, the shorter RNAemia and lower BTV detection frequency in organs of the vaccinated animals might reflect a partial NS proteins based protection. In addition, VP7 peptides have also been shown to be recognized by CTL in natural host and are considered to share similar sequences for several BTV serotypes [42]. This might be one of the underlying mechanisms that could explain the partial cross-protection of BTV1 immunization against BTV23 challenge described by Umeshappa et al. [14]. This is not contradictory to the findings of the present study, as BTV8 is as remotely related to the other European tested serotypes than BTV1 is related to BTV23, with respect to Erasmus description [11].

In our study no differences were observed among the haematology parameters measured after challenge among vaccinated and non-vaccinated groups. Monocytosis starting from 4 dpi and can be directly linked to BTV infection which induces transcriptional activation of bovine monocyte-derived macrophages [43]. The increase of monocyte count in infected cattle might be part of the mechanisms explaining the moderate clinical picture in bovine, by contrast to the severity of the disease in sheep [44]. Indeed, differences in BTV pathogenesis in sheep and cattle were reported to be related to different production levels of vasoactive mediators [44]. Simultaneous in vivo experimental infection of cattle and sheep could be carried out along with a detailed characterization of the cytokines produced in each species to clarify this hypothesis.

The minimum level of required viraemia to infect a vector has been established for several arthropod borne pathogens of major concern, like West Nile Virus [45], Dengue virus [46] or Chikungunya virus [47]. For BTV, MLVs are expected to lead to a viraemia lower than 10^3^ PFU/mL, which is about 10^3155^ TCID_50_/mL, supposed to prevent oral infection of *Culicoides* [48], even if it has been clearly reported that many MLVs could give raise to higher titres in experimental conditions and possibly lead to MLV circulation in the field in Europe [49, 50]. In the current experiment, only BTV9 and BTV16 infection could reliably reproduce a viraemia above 10^3155^ TCID_50_/mL at some points. It is however important to stress that the putative 10^3155^ TCID_50_/mL threshold is largely discussed and remains debatable. End-point titration relies on the cell-lines used and on the time of incubation before staining, both critical parameters that have no gold standard. Therefore, although useful within a study itself, direct comparison of end-point titration values from different sources would provide not much more than coarse approximation of infectivity potential. In addition, it has been reported on several occasions that vector could clearly acquire BTV from animals with a lower viraemia, sometimes even undetectable through classical isolation techniques [51, 52]. Moreover, viraemia levels only make epidemiological sense in the light of the considered serotype or strain [45] and vectors biology, i.e. biting rates and oral susceptibility, as these parameters are species dependent and likewise population dependent [53]. These results also highlight the interest of using natural host species; indeed the tested serotypes had in VERO cell culture replication properties that did not significantly differ whereas in cattle in the current study BTV1, 9 and 16 appear to be better adapted to cattle than BTV2 and BTV4. In addition, Coetzee et al. recently reported the absence of correlation between replication levels in VERO cells and virulence in ruminant host [54]. Therefore, as useful as could in vitro tests or mice models be to clarify pathogenicity mechanisms or to allow preliminary vaccine evaluations, they are unable to fully replace natural host experimental infections, which has to be chosen with special emphasis on the specie that represent the most relevant economical issue.

Broadly speaking, the results only indicated minor significant differences between the vaccinated/non-vaccinated groups although some quite obvious differences between the different serotype groups. One of the factors surely attributing to the non-significant differences is the fact the number of animals per group was low thus limiting the power of the statistical analysis. Further study focussing on some particular aspects using a higher number of individuals could clarify some of those points.

Amongst the BTV serotypes evaluated in this study, BTV1, BTV9 and BTV16 appeared to be better adapted to cattle host than BTV2 and BTV4. None of the tested serotypes could cause serious clinical disease. Viral RNA copy number was higher at viraemic peak in non-vaccinated animals. Viral detection at the viraemic peak and in the organs at necropsy suggests a partial and minor protection of BTV8 vaccination against infection with European heterologous serotypes in an experimental context. The very limited serological cross reactivity between the different tested serotypes most likely suggests cellular based mechanisms. It has been recently reported that West Nile virus lineages that induce different mortality rates in the field could cause similar mortality in experimental conditions, the discrepancy putatively thought to be linked to a different host competence among these strains [55]. Vector borne viruses are in permanent interaction with environment, hosts and vectors; therefore the epidemiological meaning of the potential effect of a mass anti-BTV8 vaccination to (partially) protect cattle livestock from heterologous serotypes remains uncertain.

## Abbreviations

BTV: bluetongue virus
BT: bluetongue disease
ELISA: enzyme linked immunosorbent assay
RTqPCR: reverse transcription quantitative polymerase chain reaction
BoHV1: bovine herpesvirus type 1
BSL3: biosafety level 3
TPI: The Pirbright Institute
CODA–CERVA: Centrum voor Onderzoek in Diergeneeskunde en Agrochemie–Centre d’étude et de recherches vétérinaires et agrochimiques
BHK: baby hamster kidney
DMEM: Dulbecco’s modified eagle medium
TCID_50_: tissue culture infectious dose 50
dpi: day post-infection
RNA: ribonucleic acid
VP7: viral protein 7
PN: percentage of negativity
SNT: seroneutralization
cDNA: complementary deoxyribonucleic acid
S2: segment 2
PFU: plaque forming unit
ANOVA: analysis of variance
NV and V: non-vaccinated and vaccinated
CPE: cytopathic effect
MLV: modified live vaccine
CTL: cytotoxic lymphocyte
VERO: verda reno
